# Shoe-stiffening inserts for first metatarsophalangeal joint osteoarthritis (the SIMPLE trial): study protocol for a randomised controlled trial

**DOI:** 10.1186/s13063-017-1936-1

**Published:** 2017-04-27

**Authors:** Shannon E. Munteanu, Karl B. Landorf, Jodie A. McClelland, Edward Roddy, Flavia M. Cicuttini, Alan Shiell, Maria Auhl, Jamie J. Allan, Andrew K. Buldt, Hylton B. Menz

**Affiliations:** 1 Discipline of Podiatry, School of Allied Health, College of Science, Health and Engineering, La Trobe University, Melbourne, VIC 3086 Australia; 2 La Trobe Sport and Exercise Medicine Research Centre, College of Science, Health and Engineering, La Trobe University, Melbourne, VIC 3086 Australia; 3 Allied Health Department, Melbourne Health, 300 Grattan Street, Parkville, VIC 3050 Australia; 4 Arthritis Research UK Primary Care Centre, Research Institute for Primary Care and Health Sciences, Keele University, Staffordshire, ST5 5BG UK; 5 Department of Epidemiology and Preventive Medicine, Monash University, Alfred Hospital, Melbourne, VIC 3004 Australia

**Keywords:** Osteoarthritis, Metatarsophalangeal joint, Hallux rigidus, Foot orthoses

## Abstract

**Background:**

This article describes the design of a parallel-group, participant- and assessor-blinded randomised controlled trial comparing the effectiveness of shoe-stiffening inserts versus sham shoe insert(s) for reducing pain associated with first metatarsophalangeal joint (MTPJ) osteoarthritis (OA).

**Methods:**

Ninety participants with first MTPJ OA will be randomised to receive full-length shoe-stiffening insert(s) (Carbon Fibre Spring Plate, Paris Orthotics, Vancouver, BC, Canada) plus rehabilitation therapy or sham shoe insert(s) plus rehabilitation therapy. Outcome measures will be obtained at baseline, 4, 12, 24 and 52 weeks; the primary endpoint for assessing effectiveness being 12 weeks. The primary outcome measure will be the foot pain domain of the Foot Health Status Questionnaire (FHSQ). Secondary outcome measures will include the function domain of the FHSQ, severity of first MTPJ pain (using a 100-mm Visual Analogue Scale), global change in symptoms (using a 15-point Likert scale), health status (using the Short-Form-12® Version 2.0 and EuroQol (EQ-5D-5L™) questionnaires), use of rescue medication and co-interventions, self-reported adverse events and physical activity levels (using the Incidental and Planned Activity Questionnaire). Data will be analysed using the intention-to-treat principle. Economic analysis (cost-effectiveness and cost-utility) will also be performed. In addition, the kinematic effects of the interventions will be examined at 1 week using a three-dimensional motion analysis system and multisegment foot model.

**Discussion:**

This study will determine whether shoe-stiffening inserts are a cost-effective intervention for relieving pain associated with first MTPJ OA. The biomechanical analysis will provide useful insights into the mechanism of action of the shoe-stiffening inserts.

**Trial registration:**

Australian New Zealand Clinical Trials Registry, identifier: ACTRN12616000552482. Registered on 28 April 2016.

**Electronic supplementary material:**

The online version of this article (doi:10.1186/s13063-017-1936-1) contains supplementary material, which is available to authorized users.

## Background

First metatarsophalangeal joint (MTPJ) osteoarthritis (OA) (*International Classification of Diseases* (ICD-10) code M20.20) is a common degenerative disorder of the foot estimated to occur in 7.8% in people aged 50 years or older, with a higher prevalence observed in women, older people, and those from lower socioeconomic backgrounds [1]. First MTPJ OA is characterised by localised pain and stiffness [2]. This condition is associated with a significant reduction in both foot-specific and general health-related quality of life [3], with 71% of people with first MTPJ OA reporting it to be disabling [1]. Increasing radiographic severity of first MTPJ OA is associated with an increased prevalence of pain, deformity and decreased joint range of motion, suggesting that it may be a progressive disorder which has an accumulative impact on surrounding structures and the load-bearing function of the foot [4].

There are structural changes (joint-space narrowing and the formation of osteophytes at the dorsal aspect) that characterise first MTPJ OA and lead to a restriction in dorsiflexion motion at the joint. This restriction in motion has been speculated to be a key factor in the development of symptoms of first MTPJ OA by causing dorsal compression of the joint during the propulsive phase of gait when the first MTPJ is required to dorsiflex [5]. In addition, the restricted joint dorsiflexion that occurs in first MTPJ OA is associated with overloading of the hallux and lesser forefoot during propulsion [6, 7], as well as a shortened step length and longer stance phase duration [8]. These changes may lead to the development of secondary musculoskeletal complaints.

The treatment goals for first MTPJ OA are to reduce pain and stiffness, as well as to prevent further degeneration of the joint [9, 10]. However, there are no evidence-based guidelines for the management of this condition. Nonetheless, nonsurgical management is recognised as the first-line therapy for this condition [11], with numerous interventions recommended, including pharmaceutical interventions, rehabilitation therapy, taping, footwear modifications and orthotic devices (such as foot orthoses or insoles) [12].

Despite the broad range of treatment options for this disorder, very few have undergone rigorous scientific evaluation [12]. Consequently, the choice of intervention for this condition is frequently a matter of trial and error, leading to increased costs and prolonged disability. The existing evidence suggests that a 4-week supervised rehabilitation therapy programme involving first MTPJ mobilisation, toe-flexor strengthening, and gait retraining alleviates symptoms of first MTPJ OA (magnitude of pain measured using a 0 to 10 pain scale reduced from 6.8 ± 1.5 to 0.4 ± 0.5) [13]. We have also found that prefabricated arch-contouring foot orthoses with a cut-out beneath the first MTPJ are equally effective as rocker-sole footwear (footwear that has a rounded sole) for reducing foot pain in people with this condition [14]. However, despite the positive symptom-modifying effects of these interventions, approximately 50% of participants either had no change or worsened [14], indicating that there is a need to further study other potential interventions for this condition.

Shoe-stiffening inserts are also commonly recommended as an intervention for first MTPJ OA [12]. Shoe-stiffening inserts are made from a thin, semi-rigid material that is placed inside the shoe with the objective of reducing the rate and magnitude of dorsiflexion at the MTPJ during the propulsive phase of gait [15, 16]. This action is speculated to reduce the symptoms of first MTPJ OA by decreasing the amount of motion and the resultant dorsal compression at the first MTPJ that occurs during propulsion [15, 16]. Indeed, there is evidence to suggest that shoe-stiffening inserts can reduce dorsiflexion of the first MTPJ during gait [17]. Importantly, there is also evidence to suggest that shoe-stiffening inserts may be effective at reducing the symptoms of first MTPJ OA. Our recent case-series feasibility study of 31 participants with first MTPJ OA found clinically worthwhile improvements in foot pain and foot-related disability at 3 months [18]. Further, 78% of participants reported that the shoe-stiffening inserts were effective. Whilst these findings are promising, there is now a need to conduct a rigorous randomised controlled trial and economic analysis to evaluate whether this simple, noninvasive and relatively low-cost intervention is effective. There is also a need to determine the effects of this intervention on foot function in this cohort to understand the potential mechanism(s) of action. Therefore, the primary aim of this project is to determine whether shoe-stiffening inserts are more effective at reducing pain of the first MTPJ in people with first MTPJ OA compared to sham shoe inserts. The secondary aims are to determine (1) whether shoe-stiffening inserts are more effective at reducing first MTPJ dorsiflexion range of motion when walking compared to sham shoe inserts and (2) whether shoe-stiffening inserts are a more cost-effective treatment for first MTPJ OA compared to sham shoe inserts.

## Methods

This study protocol has been reported using the Standard Protocol Items: Recommendations for Interventional Trials (SPIRIT) guidelines [19], and the associated checklist is included as Additional file 1.

### Design

**Fig. 1:**
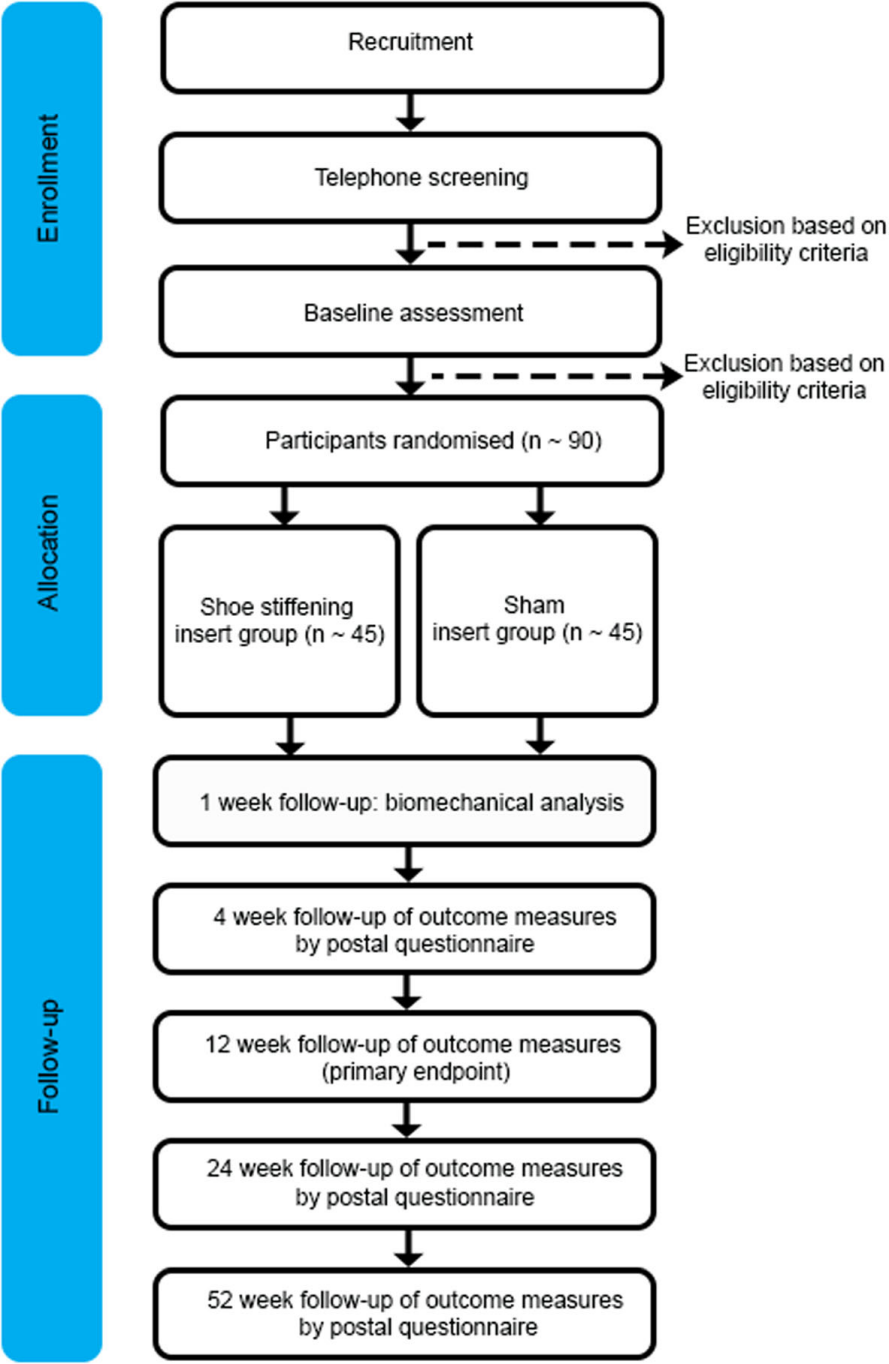
Trial profile

This study is a parallel-group, participant- and assessor- blinded, randomised controlled trial with a 52-week follow-up (Fig. 1). Participants will be randomised to an experimental group (shoe-stiffening insert(s) (Carbon Fibre Spring Plate, Paris Orthotics, Vancouver, BC, Canada) or a control group (sham shoe insert(s)). To ensure that all participants, who will have some level of pain and disability, receive some form of intervention, both groups will also be prescribed the same rehabilitation therapy programme. This design covers any ethical concerns of not treating participants in pain, but will allow the effectiveness of the shoe-stiffening inserts to be evaluated.

Due to the nature of the intervention, research staff administering the shoe insert(s) cannot be blinded to group allocation. However, research staff prescribing the rehabilitation therapy (see later), assessing outcomes and entering and analysing data will be blinded. Further, participants will be blinded to their group allocation by being informed that they will receive one of two different shoe insert treatments and a rehabilitation therapy programme for their condition.

Assessments will be performed at the La Trobe University Health Sciences Clinic, Bundoora (VIC, Australia) and the La Trobe University Gait Laboratory, Bundoora (VIC, Australia).

The study has been developed using the principles described by Osteoarthritis Research Society International (OARSI) Clinical Trials Task Force guidelines [20] and recommendations for the conduct of efficacy trials of treatment devices for OA by the Arthritis Research UK Osteoarthritis and Crystal Diseases Clinical Studies Group [21]. Publications associated with the trial will be reported according to the Consolidated Standards of Reporting Trials (CONSORT) 2010 Statement [22, 23]. The study has been registered with the Australian New Zealand Clinical Trials Registry (ACTRN12616000552482).

### Ethics approval

Ethics approval has been granted from the La Trobe University Human Ethics Committee (number HEC15-128). Informed consent will be obtained from all participants. Ethical standards will adhere to the National Health and Medical Research Council (NHMRC) National Statement [24] and the World Medical Association’s Declaration of Helsinki [25].

### Participant recruitment and eligibility criteria

Participants will be recruited by advertisements placed in local newspapers, by posters placed in senior citizens’ centres and retirement villages, mail-out advertisements to health care practitioners in Melbourne, mail-out to people currently accessing podiatry services at the La Trobe University Health Sciences Clinic, and through social networking media (e.g. Facebook, Twitter).

#### Inclusion criteria

To be included in this study, participants must: (1) be aged at least 18 years, (2) report having pain in the first MTPJ on most days for at least 12 weeks, (3) report having pain rated at least 30 mm on a 100-mm Visual Analogue Scale (VAS), (4) have pain upon palpation of the dorsal aspect of the first MTPJ and restricted first MTPJ dorsiflexion (less than 64° of dorsiflexion range of motion) [26], (5) be able to walk household distances (more than 50 m) without the aid of a walker, crutches or cane, (6) be willing to have their foot X-rayed, (7) agree to attempt to not receive additional interventions (such as shoe modifications, physiotherapy, foot orthoses, intra-articular injections, or surgery) for the first MTPJ pain during the course of the study, (8) be able to reach their feet to perform ‘rehabilitation therapy’ of the first MTPJ and (9) be willing to attempt to discontinue consuming any pain relieving medications for first MTPJ OA (except paracetamol (up to 4 g per day) which will be rescue medication) for at least 14 days prior to the baseline assessment and during the study period. Participants who consume paracetamol for first MTPJ pain prior to recruitment will be advised to discontinue its use at least 24 h prior to the baseline assessment and follow-up assessments at 4, 12, 24 and 52 weeks.

#### Exclusion criteria

Exclusion criteria for participants in this study will be: (1) previous first MTPJ surgery, (2) currently pregnant, (3) significant first MTPJ deformity including hallux valgus (defined as a score of 2 or 3 using the Manchester scale [27, 28]), (4) the presence of one or more conditions within the foot or ankle that could confound pain and functional assessments of the first MTPJ such as forefoot pain that is not first MTPJ OA, (5) the presence of any systemic inflammatory condition such as gout or rheumatoid arthritis, (6) any medical condition that, in the opinion of the investigators, makes the participant unsuitable for inclusion (e.g. clinically important pain in the musculoskeletal system other than the first MTPJ), (7) an inability to speak and read English, (8) cognitive impairment, (9) intra-articular injections (such as corticosteroids) to the first MTPJ in the previous 3 months, (10) unwilling to discontinue use of any foot orthotic devices if currently wearing them, (11) currently wearing shoe-stiffening inserts and (12) regularly wear shoes that are not able to accommodate the shoe-stiffening inserts.

### Baseline assessment

#### Participant characteristics, major medical conditions, health status and anthropometrics

Participant characteristics (such as age, sex, weight, height, education and income level), major medical conditions and number of medications will be obtained via a structured questionnaire. Health status will be measured using the Short-Form-12 (SF-12®) Version 2.0 and the EuroQol 5D-5L™ (EQ-5D-5L™) questionnaires. Height and weight will be measured using a stadiometer and digital scales and Body Mass Index will be calculated as weight (kg)/height (m^2^). Static foot posture will be assessed using the Foot Posture Index [29]. First MTPJ dorsiflexion range of motion will be measured using a reliable goniometric technique [26]. Footwear will also be assessed using selected items from the Footwear Assessment Tool [30].

#### Radiographic assessment

Participants will be required to undergo radiographic imaging of their symptomatic foot (or most symptomatic foot) to grade the presence and severity of first MTPJ OA. Dorso-plantar and lateral radiographic projections will be obtained with the participant weight-bearing in a relaxed bipedal stance position, as described previously [31]. The severity of the osteophytes and the joint-space narrowing at the first MTPJ will be determined using the *La Trobe University radiographic atlas for first MTPJ OA* [31]. All measures will be conducted by two experienced raters (SEM, HBM) who were involved in the development of the atlas. The atlas has excellent reliability [31].

### Interventions

#### Random allocation and concealment

Participants will be allocated to the intervention or control groups using minimisation [32] incorporating stratifications by age (18 to 40, 41 to 60, older than 61 years) and sex, using an interactive voice response telephone service provided by the NHMRC Clinical Trials Centre at the University of Sydney, Sydney, Australia.

#### Full-length shoe-stiffening inserts (intervention group)

**Fig. 2:**
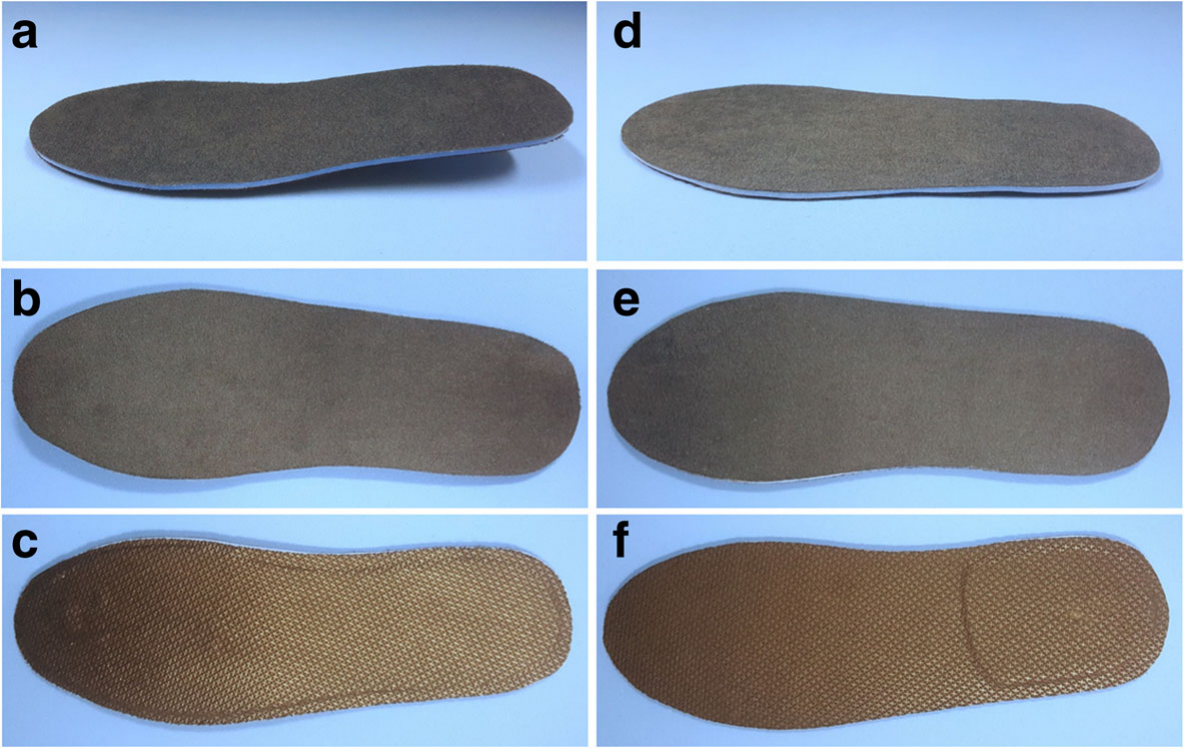
Shoe insert conditions. Panels **a** to **c** Shoe-stiffening inserts from *side*, *top and bottom view*; Panels **d** to **f** sham inserts from *side*, *top and* *bottom view*

Participants will be provided with full-length shoe-stiffening inserts at the baseline assessment (a single insert if symptoms are unilateral, or a pair of inserts if symptoms are bilateral). The shoe-stiffening inserts are light (32 to 48 g across the extra small to large size range) and thin (1.5 mm) to allow easy fitting into different types of footwear. The shoe-stiffening inserts are commercially available and are fabricated from prepregnated carbon (Carbon Fibre Spring Plate, Paris Orthotics Ltd., Vancouver, BC, Canada) with the following design characteristics: (1) full length that extends from the heel to the tip of the toes, (2) no arch build-up or contour at the heel and (3) contoured design from proximal to distal to allow for the pitch of the shoe. To maximise comfort and adherence, the inserts will be covered with 3.2-mm PPT® with an Ultralux top layer (PPT2 809 Blue, Langer Biomechanics, Ronkonkoma, NY, USA). A full-length piece of Cambrelle® (Camtex Fabrics Ltd., United Kingdom) will also be applied to the underside of the insert to make it look as similar as possible to the sham insert and to prevent abrasion prematurely wearing the undersurface of the PPT® (Fig. 2). The inserts will be dispensed by the study investigators (JJA or AKB), both registered podiatrists with a minimum 3 years of clinical experience.

#### Sham inserts (control group)

Participants allocated to the control group will receive sham inserts (a single insert if symptoms are unilateral, or a pair of inserts if symptoms are bilateral) that will be designed to not affect first MTPJ dorsiflexion but appear as identical to the shoe-stiffening inserts as possible. To achieve this, the shoe-stiffening inserts (i.e. the same as those provided to the intervention group) will be modified by removing the distal end of the insert so that the anterior edge finishes proximal to the level of the MTPJs at the anterior margin of the heel. Similar to the inserts provided to the intervention group, the sham inserts will be sandwiched between a full-length layer of 3.2-mm PPT® with an Ultralux top layer (PPT2 809 Blue, Langer Biomechanics, Ronkonkoma, NY, USA). A full-length piece of Cambrelle® (Camtex Fabrics Ltd., Cumbria, United Kingdom) will also be applied to the underside of the insert (Fig. 2). This sham insert device is necessary in this trial due to participants’ expectation of receiving a ‘take-home’ intervention (i.e. minimises resentful demoralisation). The inserts will be dispensed by JJA or AKB.

**Fig. 3:**
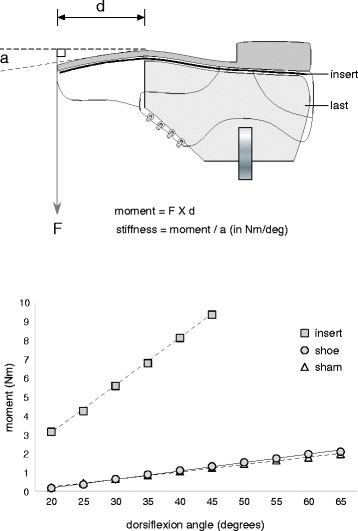
Mechanical testing of the inserts. The measurement of the bending stiffness of the inserts placed inside an Oxford shoe (*upper panel*) and the results of the stiffness of the shoe with and without a sham and full-length shoe-stiffening insert (*lower panel*). The stiffness of each condition is reflected in the gradient of the plots

Mechanical testing of the inserts has been conducted to determine their effect on the bending stiffness of the shoe. A standard Oxford shoe was affixed to a shoe last that was cut at the level of the MTPJs to provide a sagittal-plane axis. The shoe was then dorsiflexed at 5° increments (commencing at 20° of dorsiflexion), and the corresponding force was recorded using a strain gauge. The average moment obtained from an average of five repeated trials was then calculated, as illustrated in Fig. 3. The bending stiffness at 45° of dorsiflexion (the maximum dorsiflexion available with the shoe-stiffening insert placed in the shoe) for each of the conditions was as follows: shoe-only (0.027 Nm/deg), sham insert (0.029 Nm/deg) and shoe-stiffening insert (0.208 Nm/deg). These results confirm that the sham insert has a negligible effect on bending stiffness, whereas the shoe-stiffening insert results in an almost 10-fold increase in bending stiffness.

#### Rehabilitation therapy

Both groups will receive a standardised programme of rehabilitation therapy that is based on a physiotherapy programme for first MTPJ OA reported to be effective in a randomised trial [13]. This will be a home-based programme performed daily for approximately 30 min for 12 weeks, then three times per week for the remaining 40 weeks of the trial. Each therapy session will involve: (1) application of a heat pack for 10 min, followed by (2) self-mobilisation of the first MTPJ for 2 min (distraction and gliding), (3) toe-flexor strengthening exercises (one set of 10 isometric contractions each held for 10 s) and concluding with (4) application of a cold pack (10 min). If the condition is bilateral, both feet will be treated. The programme can be viewed at: https://www.youtube.com/watch?v=cHeO2H6s3K0&feature=youtu.be.

During the baseline assessment, participants will be instructed to perform the exercises by JJA or AKB. Instruction will be given prior to the shoe insert(s) being allocated and dispensed so that JJA and AKB are blinded to intervention allocation. Participants will be provided with all necessary equipment, along with an instruction booklet, so that the exercise programme can be performed unsupervised at home. The rehabilitation therapy will be reviewed at 1 and 12 weeks by research personnel (SEM, KBL or HBM), registered podiatrists with at least 19 years’ clinical experience each, blind to intervention allocation. After the 12-week review, participants will be telephoned at monthly intervals to remind them to perform the therapy (to maximise adherence). Rehabilitation therapy has been included as a co-treatment, as using a sham treatment alone may be considered withholding ‘usual’ care.

### Treatment credibility/expectation

A participant’s expectations and their initial beliefs about the credibility of a given intervention may affect the final intervention outcome [33]. Treatment credibility (participants’ beliefs about the logic underpinning the intervention) and treatment expectancy (participants’ perceptions of how much they may benefit) will, therefore, be quantified using the Credibility/Expectancy Questionnaire (CEQ) [34]. The CEQ will be administered after randomisation and allocation. The CEQ consists of six items; three are related to credibility and three are related to expectancy. For each item, participants will be asked to rate the credibility of the intervention and their expectations on a 9-point Likert scale. High scores on the scale indicate that the participant considers the intervention to be credible and expects it to be effective. The CEQ has been shown to have good internal consistency and test-retest reliability [34], and has recently been used to assess the credibility of sham dry needling [35], sham foot orthoses [36], as well as prefabricated foot orthoses and rocker-sole footwear [14] in clinical trials evaluating interventions for foot disorders.

### Outcome measures

Primary and secondary outcome measures will be collected at baseline and at 4, 12, 24 and 52 weeks. These time points have been selected as 4 weeks is considered the earliest time to detect an effect, 12 weeks is considered to be a clinically feasible time point when maximum effect would be expected and is, therefore, the primary endpoint [21, 37], and 24 and 52 weeks will allow us to determine the longer-term effects of the interventions [38]. To minimise participant burden, postal questionnaires will be used for the weeks 4, 24 and 52 assessments. To minimise loss of follow-up data, participants who do not return questionnaires will be reminded up to three times to return their questionnaires.

Biomechanical analyses will be performed at 1 week: this will minimise participant burden during the baseline assessment and enable some level of habituation to the interventions.

#### Primary outcome measure

The primary outcome measure will be the foot pain domain of the Foot Health Status Questionnaire (FHSQ) [39]. The FHSQ consists of 13 questions that assess foot health in four domains: ‘foot pain’, ‘foot function’, ‘footwear’ and ‘general foot health’. There is a total of four questions under the ‘foot pain’ domain. Questions are scored using a Likert response format and the participants’ responses are transformed into a score ranging from 0 to 100 for each domain (0 = worst foot health and 100 = optimal foot health) [39]. The FHSQ has been subjected to an extensive validation process, with each domain being shown to demonstrate high internal consistency (Cronbach’s *α* ≥0.851), good reproducibility (intraclass correlation coefficients (ICCs) ≥0.740) and discriminant validity [39], as well as good responsiveness [40]. Further, the FHSQ is rated as one of the highest-quality foot health status measures currently available [41, 42] and has been used previously in clinical trials of interventions for first MTPJ OA [14, 18, 37].

#### Secondary outcome measures

The secondary outcome measures will include:

1. Foot-related disability (using the foot function domain of the FHSQ) [39]
2. Severity of pain at the first MTPJ whilst walking over a flat surface and during rest over the past week (each via a 100-mm VAS)
3. Self-reported magnitude of symptom change (using a 15-point Likert scale where the responses range from ‘a very great deal better’ to ‘a very great deal worse’). This variable will then be dichotomised into the categories of ‘effective’ (‘a very great deal better’, ‘a great deal better’, ‘a good deal better’, ‘moderately better’) and ‘ineffective’ (‘somewhat better’, ‘a little better’, ‘about the same, hardly any better at all’, ‘no change’, ‘about the same, hardly any worse at all’, ‘a little worse’, ‘somewhat worse’, ‘moderately worse’, ‘a good deal worse’, ‘a great deal worse’, ‘a very great deal worse’) [14]
4. Level of physical activity (using the using the Incidental and Planned Activity Questionnaire (IPAQ)) [43]
5. Health status (using the Short-Form-12 Version 2 (SF-12®) questionnaire [44] and EuroQol (EQ-5D-5L™) questionnaire [45])
6. The use of paracetamol rescue medication (number of participants and mean consumption) and co-interventions to relieve pain at the first MTPJ, documented with a monthly diary throughout the 52-week study period [14, 37, 46]

### Evaluation of adherence

Adherence to the interventions (shoe inserts and rehabilitation therapy) in both groups will be assessed at monthly intervals up to 52 weeks via postal survey. For the shoe insert interventions, participants will provide information regarding the number of hours per day and number of days that they have worn their inserts during the previous 4 weeks. For the rehabilitation therapy intervention, participants will provide information regarding the average number of days per week that they have performed their exercises during the previous 4 weeks. To minimise participant burden, adherence will be documented on the day with recall over the previous 4 weeks, rather than by daily diary entries [14, 46, 47].

**Fig. 4:**
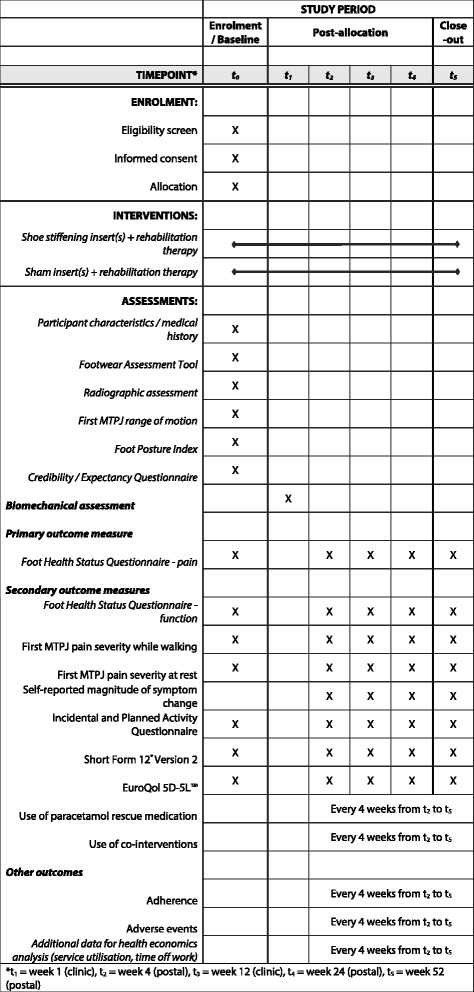
SPIRIT (Standard Protocol Items: Recommendations for Interventional Trials) diagram of enrolment, interventions and assessments for the SIMPLE trial

A summary of the data collection time points for each of the outcome measures is shown in Fig. 4.

### Adverse events

Adverse events from the shoe insert(s) and rehabilitation therapy (such as skin blistering or the occurrence of new pain or injuries in other areas of the foot and body) will be assessed at monthly intervals up to 52 weeks via postal survey. Participants will be asked to document the type of adverse event, the body location, the frequency and/or severity of the event [14, 46, 47]. If participants experience more significant adverse events (e.g. severe pain), they will be advised to contact one investigator who is not involved in recruitment, allocation or data collection (SEM, KBL, HBM). All adverse events will be reported in the final manuscript.

### Biomechanical evaluation

**Fig. 5:**
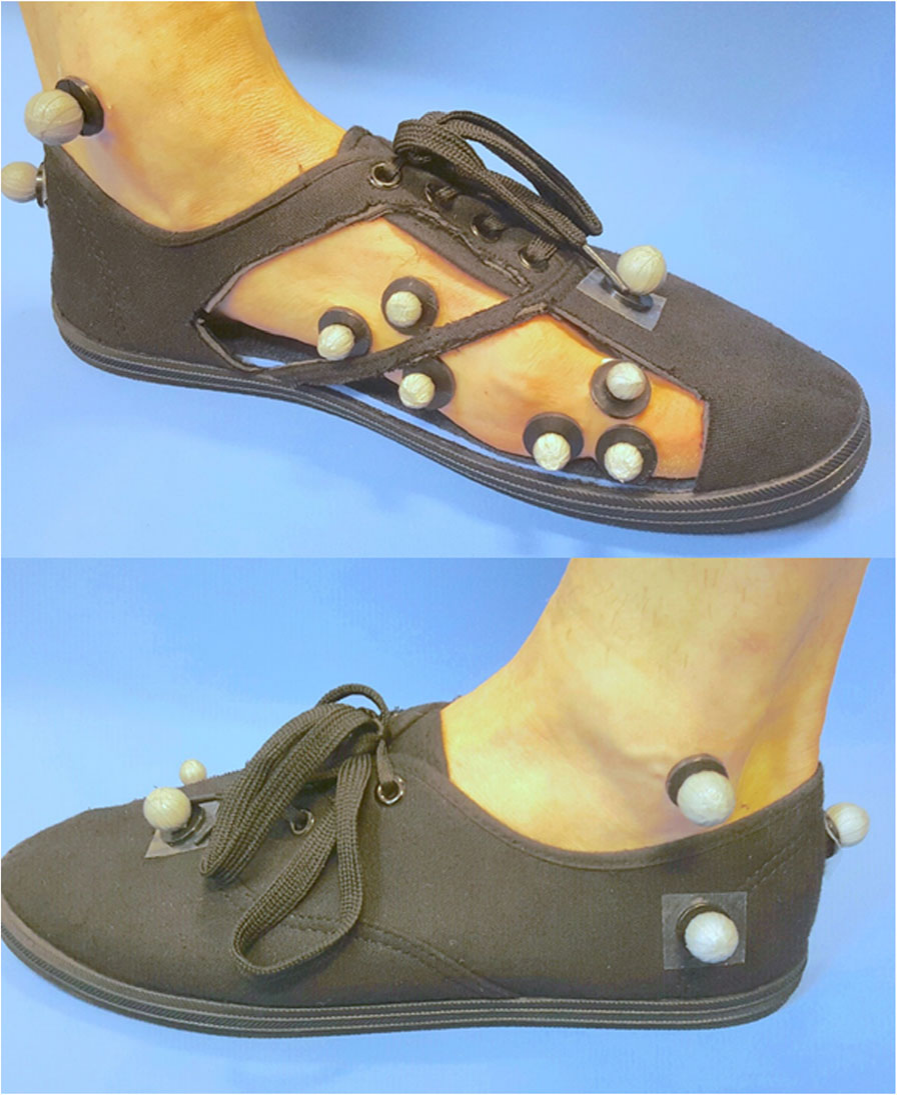
Marker placement for measurement of first metatarsophalangeal joint (MTPJ) kinematics. The effect of the inserts on first MTPJ kinematics will be performed using the Salford Foot Model with participants wearing ‘gait shoes’ that have been customised with cut-outs of the upper in order to allow clear visualisation of the foot markers. *Upper panel*: medial view; *Lower panel*: lateral view

Biomechanical evaluation will be performed to evaluate change in biomechanical function of the first MTPJ using the shoe-stiffening inserts as this is the proposed mechanism of action of the shoe-stiffening inserts. Measurement of first MTPJ kinematic variables (range of motion of first MTPJ and maximum first MTPJ dorsiflexion) will be performed during level walking, in addition to kinematic and kinetic analysis of the hip, knee and ankle. These assessments will be performed at 1 week, rather than baseline, to minimise participant burden and allow for some level of habituation [36]. Three-dimensional kinematics will be measured using a 10-camera infrared motion analysis system (Vicon Motion Systems Ltd., Oxford, UK). Two force plates (Kistler, 9865B, Winterthur, Switzerland and AMTI OR6, Watertown, MA, USA) will be used to identify gait cycle events and record kinetic data. Six passive retro-reflective markers will be attached to the medial forefoot (three markers) and proximal phalanx of the hallux (three markers) as required for calculation of first MTPJ kinematics using the Salford Foot Model [48]. The Salford Foot Model is a validated five-segment model that allows calculation of the kinematics of the calcaneus relative to the tibia, the midfoot relative to the calcaneus, the lateral forefoot and medial forefoot segments relative to the midfoot, and the hallux relative to the medial forefoot. In this study, the kinematics of the hallux relative to the medial forefoot will be focussed on in the analysis. This model has been used to assess first MTPJ kinematics with acceptable reliability [49]. Marker trajectories (100 Hz) and force plate (4000 Hz) data will be collected synchronously using Vicon Nexus software (Vicon Motion Systems Ltd., Oxford, UK). In addition, 32 markers will be fixed to anatomical landmarks of the trunk, pelvis and lower limb based on the modified Helen Hayes marker set [50, 51], as well as a customised model to allow for segmental definition and functional joint calibration. All lower-limb joint kinematics will be calculated based on Euler angles and described in terms of movement of the distal segment relative to the proximal segment. Data will be collected and averaged from the middle stride of six 10-m walking trials for each condition. Comparisons between intervention groups will be made for differences with and without the intervention (shoe-stiffening insert or sham insert) with participants wearing ‘gait shoes’ (canvas ‘Now’ and ‘IND99’ shoes, Kmart Australia – shoes are the same but have different branding), which comprise a laced fastening and canvas upper, and are customised with cut-outs of the upper in order to allow clear visualisation of the foot markers (Fig. 5). It is not possible to perform kinematic analysis of foot function using participants’ own closed-in shoes due to the need to attach retro-reflective markers to the foot landmarks. These analyses will be adequately powered (power >0.8 assuming *n* ≥30 and standard deviation (SD) = 7.5 [8]) to detect a difference of 5.5° in first MTPJ dorsiflexion range of motion between the interventions.

### Economic evaluation

The economic analysis will take a health service perspective, assessing the difference in health sector costs and comparing this with the difference in health outcomes between treatment and comparison groups. Participants’ time costs will be similar between both groups and do not affect the comparison. Collection of health service resource use will be informed by the Australian Guidelines for Preparing Submissions to the Pharmaceutical Benefits Scheme [52]. Participants will be asked to document their health service use, use of co-interventions, days unable to work and impairments in physical activity due to first MTPJ OA in the 4 weeks prior to each survey round (monthly intervals up to 12 months). Service encounters (e.g. GP consultations) will be converted to costs using the Pharmaceutical Benefits Advisory Committee (PBAC) Manual of Resource Items [53]. Both the cost-effectiveness and the cost-utility of the intervention will be determined: the latter as it allows comparisons of economic value with a broader range of health service interventions. The cost-effectiveness analysis will estimate the additional (or incremental) cost per person achieving a meaningful improvement in the FHSQ pain score (i.e. larger than the minimal important difference). The cost-utility analysis will use the results from the EQ-5D-5L™ to generate a health-related quality of life utility score at baseline and trial follow-up, which will be used to estimate change across the two groups. From this, an incremental cost per quality-adjusted life year will be derived. Probabilistic sensitivity analyses will be conducted, incorporating confidence limits on the plausible range of costs and effects used in the analysis.

### Sample size

The sample size has been determined a priori using SPSS Sample Power 3.0 (IBM Corp., Armonk, NY, USA) based on the FHSQ pain domain as the primary outcome measure. Using a power of 90%, minimal important difference of 12.5 points in the foot pain domain of the FHSQ [37, 54], SD of 16.8 (based on the 12-week time point in our recent trial [14]), assuming a 10% dropout rate, and a significance level set at *α* < 0.05, we estimated that a total of 90 participants will be required.

### Statistical analysis

Statistical analysis will be performed using the most recent version of SPSS (IBM Corp., Armonk, NY, USA) available at the time of analysis. Analysis will adhere to the intention-to-treat principle for all randomised participants. In participants with bilateral symptoms, the more painful foot will be analysed (or the right foot if they cannot define the more painful foot) to maintain independence of data. Multiple imputation will be used to replace any missing data using five iterations, with sex, age, baseline scores and group allocation as predictors. The exception will be for the following variables where no data substitution will be applied: self-reported magnitude of symptom change, use of co-interventions, and adverse events. Standard tests to assess continuous data for normal distribution will be used and transformation carried out if required. The primary outcome measure will be the foot pain domain of the FHSQ measured at 12 weeks. To avoid over-testing and to minimise the risk of type I error associated with serial measurements, statistical analysis of the effectiveness of the interventions will specifically focus on the change in primary outcome measures between baseline and 12 weeks [55, 56], and differences in the primary and secondary outcome measures between the two groups will be compared at 12 weeks. Continuously scored outcome measures (FHSQ foot pain domain, FHSQ foot function domain, severity of pain at the first MTPJ whilst walking over a flat surface and during rest in the previous week, level of physical activity using the IPAQ, and health status using the SF-12® and EQ-5D-5L™) will be analysed using analysis of covariance with the intervention group and baseline scores entered as independent variables. Self-reported magnitude of symptom change will be compared using relative risk, risk difference and number needed to treat (NNT). Use of paracetamol rescue medication and intervention adherence will be compared using independent groups *t* tests. Use of co-interventions and frequency of adverse events will be compared using relative risk and risk difference statistics. For the biomechanical evaluation, first MTPJ range of motion and maximum first MTPJ dorsiflexion will be compared between groups using analysis of covariance with the intervention group and shoe-only condition scores entered as independent variables.

## Discussion

Osteoarthritis of the first MTPJ is highly prevalent and causes significant pain and disability in those affected. Developing effective interventions for first MTPJ OA is, therefore, a high priority. At present, there is little evidence to guide management of this condition as, to our knowledge, there are only three clinical trials that have been published [13, 14, 37]. Consequently, the choice of intervention is currently a matter of trial and error, leading to increased costs and prolonged disability. We will address this issue by conducting a rigorous randomised controlled trial and economic analysis to evaluate the effectiveness of shoe-stiffening inserts for reducing foot pain, improving mobility and health-related quality of life in people with first MTPJ OA.

In this trial, both intervention groups will receive a standardised programme of rehabilitation therapy that is based on a physiotherapy programme for first MTPJ OA which has been reported to be effective in a randomised trial [13]. The therapy will be performed daily for the initial 12 weeks then three times per week for the remainder of the trial (up to 52 weeks). The programme to be used in this trial is similar to the previous trial [13] in that it involves mobilisation of the first MTPJ and strengthening of the plantar-flexor muscles of the first MTPJ. However, it does differ from the previous trial [13] in a number of ways: (1) it is primarily a home-based programme rather than a clinician-administered programme, (2) the duration will be 52 weeks rather than 4 weeks, (3) it will be performed daily during the initial 12 weeks, rather than three times per week, (4) a heat pack will be used to increase soft tissue extensibility at the start of each session rather than a hot whirlpool bath and therapeutic ultrasound, (5) mobilisation of the first MTPJ will involve the first metatarsal and proximal phalanx (and will not include sesamoid mobilisation), (6) gastrocnemius and hamstring muscle stretching will not be performed, (7) active plantarflexion of the ankle joint and a marble pick-up exercise with the toes will not be performed, (8) ‘gait training’ will not be prescribed and (9) a cold pack rather than electrical stimulation will be used at the conclusion of each session. We have amended the programme because (1) we believe that elements of the programme in the previous trial [13] are unlikely to be of clinical benefit in first MTPJ OA, (2) the programme will need to be performed for 52 weeks rather than 4 weeks, so minimising participant burden is important and (3) it will primarily be a home-based programme, so needs to be simple to perform. Importantly, a rehabilitation therapy programme has been included as a co-treatment as an ethical requirement, since using a sham treatment alone could be considered withholding ‘usual’ care.

Poor adherence by participants to their shoe insert intervention and/or rehabilitation therapy programme has the potential to confound the study results. In our previous case series study, adherence to the shoe-stiffening inserts was excellent, with the shoe-stiffening inserts being reported to be worn for a mean of 42 h per week at 3 months [18]. Furthermore, in a previous trial investigating the effectiveness of a 4-week clinician-administered rehabilitation therapy programme for first MTPJ OA by Shamus et al. [13], adherence was reported to be 100%. Therefore, we anticipate that adherence to our interventions will be satisfactory in the short to intermediate term in our trial (12 weeks). Participant adherence may reduce over the course of this trial given its extended duration (52 weeks). However, to maximise adherence, participants will be (1) telephoned at monthly intervals to remind them to perform the rehabilitation programme and (2) asked to complete diary entries regarding the use of their shoe inserts and completion of the rehabilitation programme that will be returned at monthly intervals.

This trial will incorporate five repeated measurements for the primary outcome measure (baseline, 4, 12, 24 and 52 weeks) to provide insights into the trajectory of any improvements in symptoms. The three previous clinical trials investigating nonsurgical interventions for first MTPJ OA have used study durations of 4 [13], 12 [14] and 24 [37] weeks. Incorporating a 52-week follow-up period is a major strength of our study as it will enable us to determine the short- (4 weeks), intermediate- (12 weeks) and longer-term (24 and 52 weeks) effects of the shoe-stiffening inserts, if any. A 52-week follow-up is also important to allow the capture of important costs and effects for the planned economic analysis [57].

This will be the first trial to perform an economic analysis of any nonsurgical intervention for first MTPJ OA. Inclusion of such an analysis is important because the acceptance and use of novel interventions is determined by their cost as well as by their clinical effectiveness [58]. Results from this study will, therefore, be of value to both clinicians and health care policy-makers. Recruitment of participants will commence in April 2016 and final results are expected to be available in December 2018.

## Trial status

Advertising for participants commenced in April 2016 with the first participant being enrolled on 16 June 2016. The final results are expected to be available in December 2018.

## Additional file

Additional file 1: SPIRIT (Standard Protocol Items: Recommendations for Interventional Trials) Checklist for the SIMPLE trial. (DOC 121 kb)

## Abbreviations

CEQ: Credibility/Expectancy Questionnaire
CONSORT: Consolidated Standards of Reporting Trials
EQ-5D-5L™: EuroQol 5D-5L™
FHSQ: Foot Health Status Questionnaire
ICC: Intraclass correlation coefficient
*ICD*: *International Classification of Diseases*
IPAQ: Incidental and Planned Activity Questionnaire
MTPJ: Metatarsophalangeal joint
NHMRC: National Health and Medical Research Council
NNT: Number needed to treat
OA: Osteoarthritis
OARSI: Osteoarthritis Research Society International
PBAC: Pharmaceutical Benefits Advisory Committee
SF-12®: Short-Form-12
SPIRIT: Standard Protocol Items: Recommendations for Interventional Trials
VAS: Visual Analogue Scale
